# A Case of Amyloid Beta-Related Angiitis: Rare Inflammatory Vasculopathy of the Central Nervous System

**DOI:** 10.7759/cureus.88996

**Published:** 2025-07-29

**Authors:** Sai Varun Bethina, Asish Gulati, Tamra I Ranasinghe

**Affiliations:** 1 Department of Neurology, Virginia Commonwealth University School of Medicine, Richmond, USA; 2 Department of Neurology and Rehabilitation Medicine, George Washington University Hospital, Washington, DC, USA; 3 Department of Neurology, Mayo Clinic, Phoenix, USA

**Keywords:** abra, cerebral amyloid angiopathy (caa), cerebral amyloid angiopathy-related inflammation, cerebral vasculopathy, primary angiitis of the central nervous system (pacns)

## Abstract

Cerebral amyloid angiopathy (CAA) is associated with amyloid-beta deposition in cerebrovascular vessels, leading to spontaneous intracerebral hemorrhage (ICH). A rare manifestation, amyloid beta-related angiitis (ABRA), presents with symptoms including subacute progressive headaches, cognitive decline, and focal neurological deficits. This case report discusses a 78-year-old female patient with a history of hypertension who exhibited symptoms consistent with ABRA, including headaches and memory impairments. Diagnostic imaging revealed significant MRI findings of confluent hyperintensities and microhemorrhages, while a temporal brain biopsy confirmed granulomatous arteritis linked to amyloid angiopathy. Treatment with corticosteroids resulted in notable clinical improvement. This report underscores the importance of early diagnosis and intervention in ABRA, promoting the establishment of non-invasive diagnostic criteria to distinguish it from other CAA-related conditions, thereby potentially facilitating prompt treatment without the necessity for invasive procedures.

## Introduction

Cerebral amyloid angiopathy (CAA) is characterized by amyloid-beta deposition in the media and adventitia of small and medium-sized cerebrovascular vessels and is a major cause of spontaneous intracerebral hemorrhage (ICH). Amyloid beta-related angiitis (ABRA) is a rare form of central nervous system (CNS) vasculitis seen in patients with CAA. Though the exact etiology of ABRA is unknown, it is postulated to be caused by a destructive inflammatory reaction to amyloid-beta fibril deposition within the arterial walls of small and medium-sized vessels, primarily located within the leptomeninges and cerebral cortex [1,2].

The most common clinical presentation of ABRA involves subacute progressive headaches, cognitive impairment, seizures, hallucinations, and focal neurological deficits [1-4]. There is a clinical overlap amongst the inflammatory CAA (I-CAA), ABRA, and primary angiitis of the CNS (PACNS). A cerebral biopsy is the gold standard for diagnosis. This case report illustrates the importance of early diagnosis and treatment of patients with ABRA.

## Case presentation

A 78-year-old female patient with a history of hypertension, depression, and osteoarthritis presented with headaches and memory difficulties. Her headaches were bi-occipital, 6-7/10 in intensity, dull in nature, and associated with dizziness, without typical migrainous features. The patient denied any change in the nature of the headache or intensity with postural changes or the Valsalva maneuver. Her headaches started five to six weeks prior to presentation and progressively worsened. Further, she complained of difficulties with her cognition and memory, especially short-term memory, for one year prior. She reported misplacing items and forgetting the names of her friends, but managed to carry out activities of daily living without any assistance. She denied any episodes suggestive of seizures. On initial assessment, she was alert and oriented to time, place, and person. On memory testing, she was able to recall only one out of three words in five minutes. She had no aphasia; her cranial nerves were intact, and she demonstrated full strength throughout, with intact sensation to light touch. Gait examination revealed an unsteady, wide-based gait, with a slow pace and a delay in turning. She had no ataxia on finger-to-nose or heel-to-shin testing but was noted to have a positive Romberg sign. 

**Figure 1:**
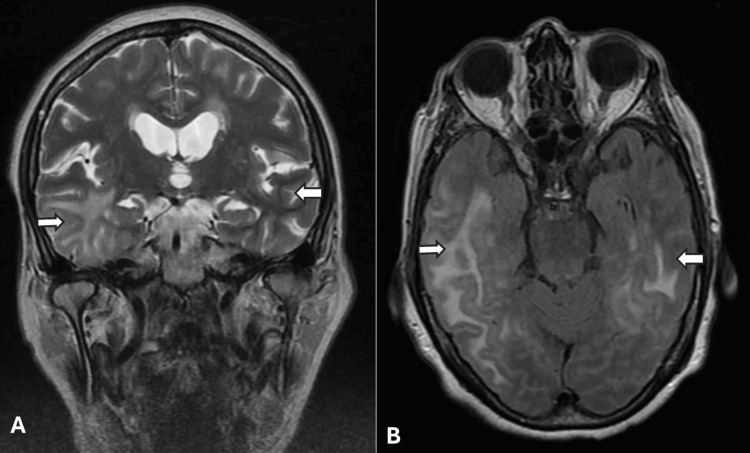
Initial MRI of the brain T2 coronal (A) and T2 FLAIR (B) images demonstrating extensive white matter hyperintensities and vasogenic edema throughout bilateral temporal lobes, the right side greater than the left side. MRI: magnetic resonance imaging; FLAIR: fluid-attenuated inversion recovery

**Figure 2:**
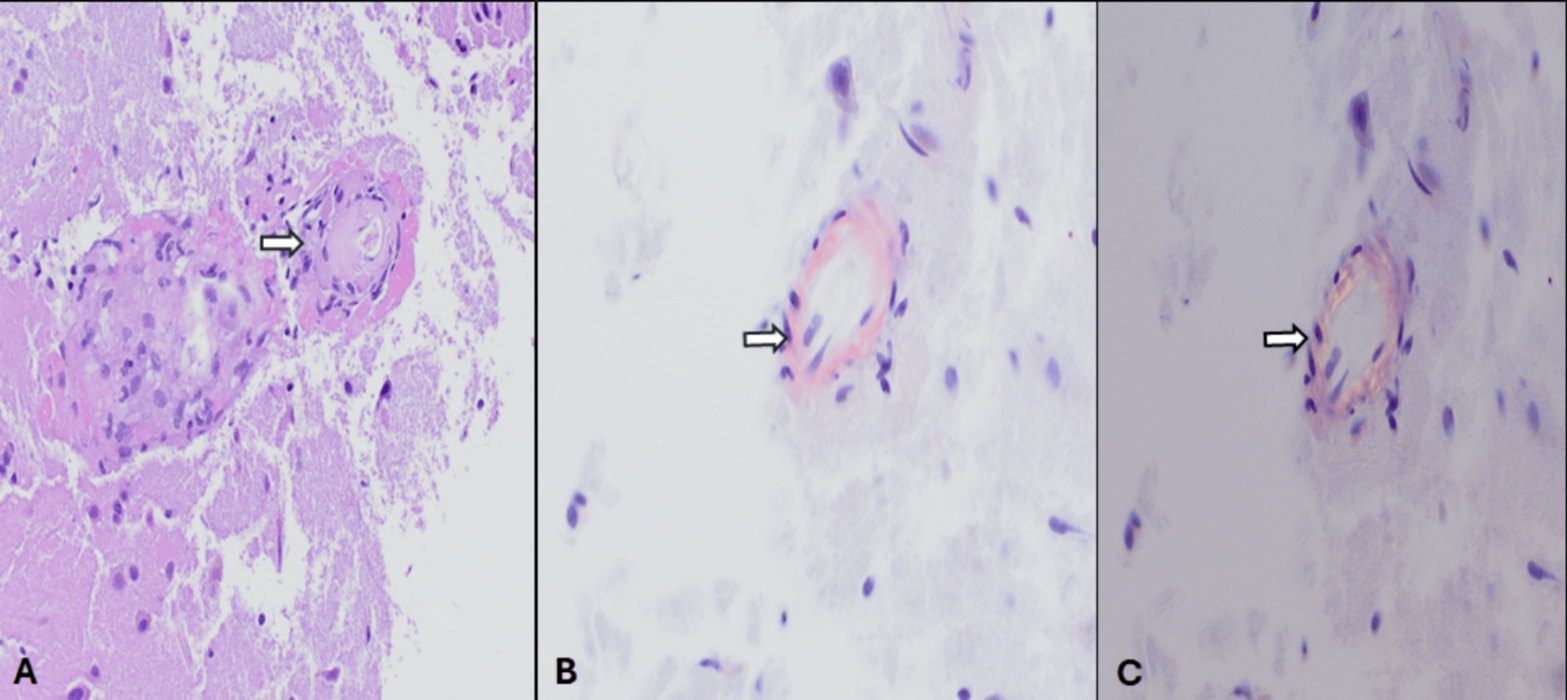
(A): Hematoxylin and eosin staining demonstrating a cortical vessel with granulomatous inflammation and amorphous eosinophilic material within the vessel wall; (B): Congo red stain positive for amyloid in an inflamed vessel; (C): Polarized Congo stain redemonstrating amyloid deposition.

**Figure 3:**
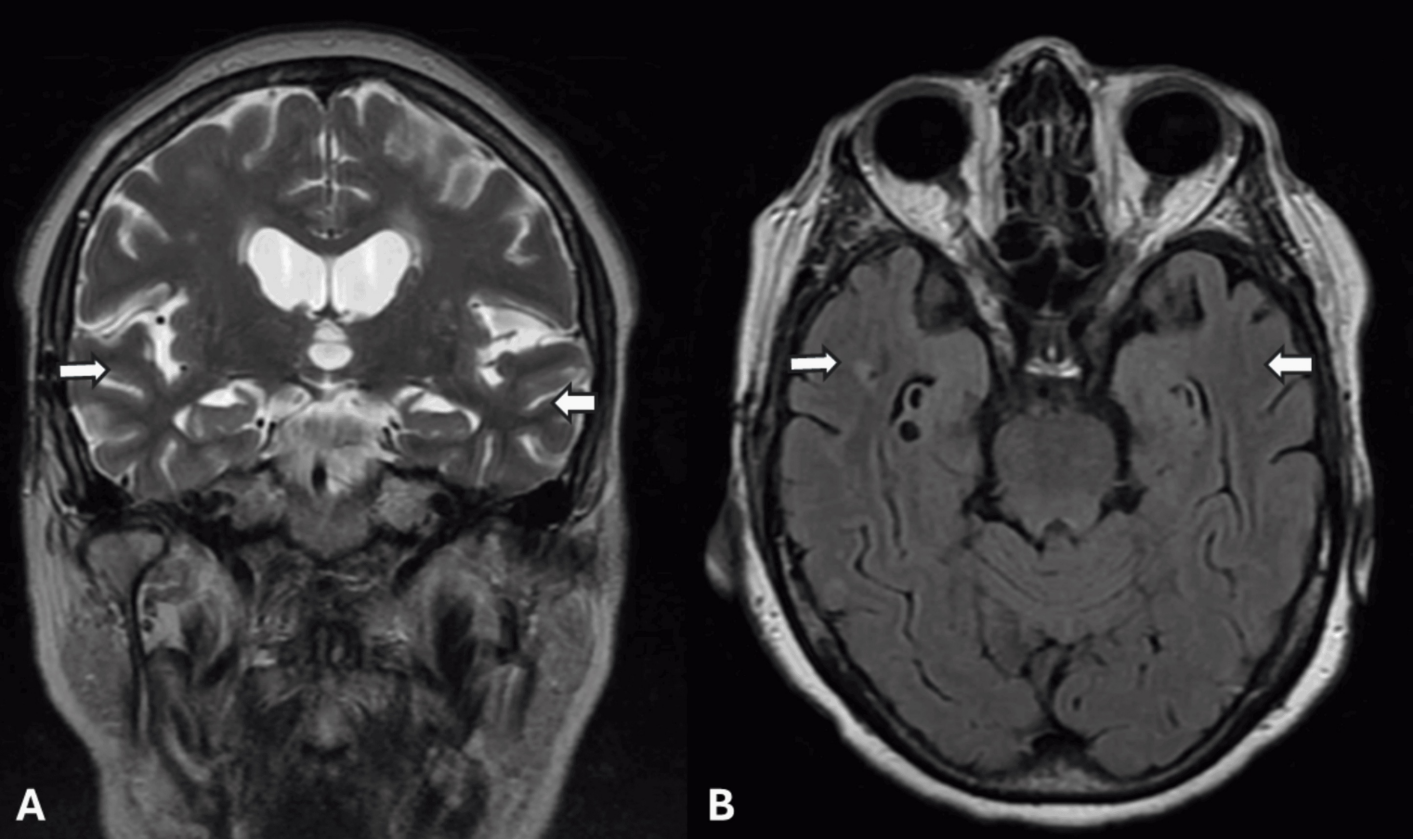
Follow-up MRI of the brain; T2 coronal (A) and T2 FLAIR (B) images demonstrating resolution of T2 FLAIR hyperintensities and vasogenic edema involving bilateral temporal lobes MRI: magnetic resonance imaging; FLAIR: fluid-attenuated inversion recovery

Her routine serum lab work, including a complete blood count, basic metabolic panel, erythrocyte sedimentation rate (ESR), and C-reactive protein (CRP), was within normal limits. Non-contrasted computed tomography (CT) of the head was unremarkable for any acute intracranial abnormalities, but magnetic resonance imaging (MRI) of the brain with and without contrast demonstrated confluent T2/fluid attenuation inversion recovery (FLAIR) hyperintensities throughout the subcortical white matter of the bilateral occipital, posterolateral temporal, and posterior parietal lobes, without any parenchymal or leptomeningeal enhancements (Figure 1). Susceptibility weighted imaging (SWI) demonstrated numerous lobar microhemorrhages concerning probable CAA. MR angiogram of the head and neck revealed moderate narrowing of the distal M1 segment of the right middle cerebral artery (MCA) without evidence of any other significant flow-limiting stenosis. Further evaluation by means of a digital subtraction angiography (DSA) showed no evidence of large or medium vessel flow-limiting stenosis or vasculitis. A lumbar puncture demonstrated cerebrospinal fluid protein 82 mg/dL, glucose 52 mg/dL, white blood cells (WBC) 7 cells/µL, and red blood cells (RBC) 1158 cells/µL (Table 1). Viral and fungal studies were negative. Flow cytology and cytometry were negative for malignancy. Given the broad differential, including ABRA, I-CAA, and PACNS, a right temporal brain biopsy was performed, which revealed granulomatous arteritis associated with amyloid angiopathy, confirming a diagnosis of ABRA (Figure 2). The patient was treated with intravenous Solu-Medrol for three days and continued on a long oral prednisone taper at discharge. On follow-up at four months, her cognition and headaches resolved, and a repeat MRI of the brain without contrast demonstrated near complete resolution of the confluent T2/FLAIR hyperintensities throughout the subcortical white matter (Figure 3).

**Table 1:** The laboratory values of the patient with reference values CSF: cerebrospinal fluid

CSF analysis	Patient's value	Normal reference value
1. Protein	82 mg/dL	15-45 mg/dL
2. Glucose	52 mg/dL	50-80 mg/dL
3. White blood cell (WBC) count	7 cells/µL	0-5 cells/µL
4. Red blood cell (RBC) count	1158 cells/µL	0 cells/µL

## Discussion

CAA-related inflammation (CAA-RI) includes two pathological subtypes: I-CAA, which is identified by perivascular inflammation around the vessel wall, and ABRA, which is identified by transmural or intramural granulomatous inflammation and destruction of the vessel wall leading to CNS vasculitis [3,5]. ApoE ɛ4 genotype is more likely to be found in I-CAA, while vessel destruction with inflammation is found in ABRA and PACNS [3,5]. However, the terms “CAA-RI,” “I-CAA,” “ABRA,” and “CAA associated with inflammation” are often used interchangeably and hence can be confusing [6]. It should be noted that some authors refer to the two pathological subtypes mentioned earlier as CAA-RI and ABRA [7,8].

ABRA is a rare inflammatory CNS vasculopathy that has clinical and neuroradiological features similar to I-CAA and PACNS. A cerebral biopsy remains the gold standard for the diagnosis of ABRA with characteristic histopathological features like frank vessel wall inflammation with destruction of the vessel wall by leucocytic infiltration forming multinucleated giant cells forming either granulomas or fibrinoid necrosis, or both, resulting from the antigenic response against the amyloid β deposits [3]. Although considered safe, stereotactic brain biopsy can result in complications with a mortality rate of up to 1% and morbidity rate of up to 5%, with postoperative hemorrhage being the most common complication [9]. If a biopsy is performed, it should include leptomeningeal, superficial cortical vessels, and gray-white junction tissue, as it is not unusual to have a non-diagnostic result if only the white matter is biopsied, which can further lead to a delayed diagnosis and treatment [10]. Further, biopsy sampling errors can occur due to the patchy nature of the inflammatory response. Given these limitations and the invasive nature of a cerebral biopsy, there have been several investigations into creating noninvasive diagnostic criteria to differentiate between ABRA, I-CAA, and PACNS.

One proposed diagnostic criterion primarily included clinical features and MRI brain imaging data. In an observational study by Salvarani et al., leptomeningeal enhancement was found on the MRI scans of the brain in 70.4% of patients with ABRA or I-CAA in comparison to 7.4% of patients with CAA. Overall, evidence of leptomeningeal enhancement on MRI was noted to have a sensitivity of 70% and specificity of 93% for the presence of vascular inflammation [11]. In contrast, patients with CAA without evidence of vascular inflammation were more likely to have an intracerebral hemorrhage on MRI with a sensitivity of 63% and specificity of 93%. Similarly, in a study by Eng et al., 33 of 35 patients with non-inflammatory CAA presented with a hemorrhagic stroke in comparison to the I-CAA patients, who more commonly presented with subacute encephalopathy or seizures [1]. Thus, the presence of leptomeningeal enhancement in the absence of an intracerebral hemorrhage increases the likelihood of CAA with vascular inflammation [1, 11]. However, this was not the case in our patient, as her brain MRI did not demonstrate any leptomeningeal enhancement.

In another study by Auriel et al., clinical and imaging criteria meeting a diagnosis of probable CAA-RI resulted in 82% sensitivity and 97% specificity, while defined criteria meeting possible CAA-RI resulted in a sensitivity of 82% and specificity of 68% [12]. The defined diagnostic criteria used in the study for probable CAA-RI included the following: (1) age greater than 40 years; (2) brain MRI with asymmetric unifocal or multifocal white matter lesions within the corticosubcortical or deep white matter; (3) presence of at least one of the common presenting symptoms, including headache, decrease in consciousness, behavioral change, focal neurological deficit, or seizures; (4) presence of at least one corticosubcortical hemorrhagic lesion, including a cerebral macrobleed, cerebral microbleed, or cortical superficial siderosis; and (5) the absence of other possible etiologies. The criteria for possible CAA-RI largely involved the same criteria, except for MRI findings with white matter lesions extending to the subcortical white matter [12]. The less restrictive criteria for the white matter patterns used in the diagnostic criteria for possible CAA-RI resulted in a lower specificity. Given the higher sensitivity and specificity of the diagnostic criteria proposed for probable I-CAA, it is possible that with further validation, these criteria can be used in identifying patients who can be treated empirically without having to undergo an invasive cerebral biopsy [12]. Utilizing the criteria proposed by Auriel et al., our patient can be diagnosed with probable CAA-RI. However, this does not further differentiate between the pathological subtypes of ABRA or I-CAA. Histopathological tissue diagnosis remains the only method to distinguish between these 2 varieties [12]. Nonetheless, there were multiple instances where patients were treated without biopsy when MRI findings were characteristic [13-15]. Recently, Charidimou et al. have also proposed a pragmatic diagnostic framework with revised criteria for CAA-RI, which may further help avoid the need for invasive biopsy in carefully selected patients presenting with typical clinical and radiological features [16]. Biopsy is preferred in either atypical presentation or patients who do not respond to treatment [17, 18].

Though early diagnosis of ABRA through histopathological examination can aid in distinction from CAA, I-CAA, and PACNS, the invasive nature of a cerebral biopsy may be avoided if diagnostic criteria are met through clinical and imaging features. If indicative of an inflammatory process, early empiric treatment with immunosuppressive therapy such as high-dose corticosteroids followed by oral steroids or cyclophosphamide can be initiated to decrease the morbidity and mortality associated with these disease processes [4, 5, 9, 19]. Close follow-up in the outpatient setting will be necessary to monitor the responsiveness to immunosuppressive therapy and the need for further diagnostic testing.

## Conclusions

This case report highlights the importance of early diagnosis and treatment of ABRA, a rare but serious CNS vasculitis associated with CAA. While cerebral biopsy remains the gold standard for diagnosis, non-invasive imaging and clinical criteria are being developed to aid in distinguishing ABRA from related conditions like I-CAA and PACNS. Additionally, a pragmatic diagnostic framework with revised criteria for CAA-RI has been recently proposed, which could potentially avoid invasive procedures for the diagnosis in such patients in the future. In our patient, early histopathological diagnosis allowed for prompt treatment with corticosteroids, leading to significant improvement in symptoms and radiological findings. As diagnostic criteria become more refined, timely intervention may increasingly be possible without the need for invasive biopsies, reducing risks and improving patient outcomes. Close monitoring and follow-up remain critical for long-term management and recovery. 
